# Explicit integration of dispersal-related metrics improves predictions of SDM in predatory arthropods

**DOI:** 10.1038/s41598-020-73262-2

**Published:** 2020-10-07

**Authors:** Jérémy Monsimet, Olivier Devineau, Julien Pétillon, Denis Lafage

**Affiliations:** 1 Department of Forestry and Wildlife Management, Inland Norway University of Applied Sciences, Campus Evenstad, Koppang, Norway; 2 UMR CNRS 6553 ECOBIO, Université de Rennes, Rennes, France; 3 Department of Environmental and Life Sciences/Biology, Karlstad University, Karlstad, Sweden

**Keywords:** Climate-change ecology, Conservation biology, Ecological modelling

## Abstract

Fishing spiders (*Dolomedes* spp.) make an interesting model to predict the impact of global changes because they are generalist, opportunistic predators, whose distribution is driven mostly by abiotic factors. Yet, the two European species are expected to react differently to forthcoming environmental changes, because of habitat specialization and initial range. We used an original combination of habitat and dispersal data to revisit these predictions under various climatic scenarios. We used the future range of suitable habitat, predicted with habitat variables only, as a base layer to further predict the range or reachable habitat by accounting for both dispersal ability and landscape connectivity. Our results confirm the northward shift in range and indicate that the area of co-occurrences should also increase. However, reachable habitat should expand less than suitable habitat, especially when accounting for landscape connectivity. In addition, the potential range expansion was further limited for the red-listed *D. plantarius,* which is more of a habitat specialist and has a lower ability to disperse. This study highlights the importance of looking beyond habitat variables to produce more accurate predictions for the future of arthropods populations.

## Introduction

Climate change, which is now threatening all ecosystems worldwide^1^, is a multi-factor problem that goes beyond raising temperatures only^2,3^. Tackling this complexity requires that ecologists obtain realistic predictions of how species distributions will change in response to global change. A poleward range shift of the distribution is expected in all continents and was observed in different taxa^4–6^. The ability to shift can nonetheless be limited for species with limited dispersal abilities or specialist species^7^. In recent years, species distribution models (SDMs) proved to be an important tool to predict geographic distributions by correlating species occupancy to environmental variables^8^. Applications include conservation planning^9^, potential invasion range^10^, or forecasting in time^11^. SDMs were successfully applied to a large variety of terrestrial (see Hao et al.^12^ for a review) and marine organisms (see Melo-Merino et al.^13^ for a review).

The accuracy of predictions produced by SDMs varies from algorithm to algorithm, even when considering that the MaxENT algorithm is most often used^14^. This variation in accuracy can be alleviated with ensemble models, which combine algorithms and produce consensual predictions^15,16^. Of course, input data also influence the predictions^17^, and while most SDMs use only climatic variables, including other variables such as land-use might improve predictions^18^. In order to make projections in time, it is fundamental to carefully select the right climatic scenario^17^. Right now, the ones produced and updated by the Intergovernmental Panel on Climate Change^19^ are the most widely recognized and used climatic scenarios.

SDMs assume that the species and its environment are at equilibrium^20^, so that all suitable locations are occupied. SDMs also assume that the ecological niche is stable, i.e. that the same factors limit the species in space and time^21^. Under these assumptions, SDMs are used to define habitat suitability, which is the range of physical locations where one species can live^22^. However, a properly constructed and calibrated SDM can provide information about the species’ realized niche, i.e. a combination of habitat with other biotic and abiotic factors^20,23^.The gold standard of SDMs would be fully mechanistic models which were used, for example to study seed dispersal in birds^24^ or population dynamics and evolution of dispersal trait^25^. However, these models are very data-demanding, and simpler hybrid mechanistic-correlative models are often more suitable for less well-studied taxa. In particular these hybrid models allow including active biological processes such as dispersal^26^. Examples include making predictions under full /no dispersal^27^ or using a buffer of dispersal around each presence^28^.

As generalist predators, spiders are relatively independent of a specific prey community, and their assemblage and distribution are mostly influenced by habitat and land use^29^, which makes them good study cases for SDMs. Fennoscandia is a potential climatic refugium for spider populations against the current global warming as their range is expected to expand Northward in Europe^30,31^. Refugia can mitigate the effects of climate change by providing suitable conditions for species persistence through time^32^. *Dolomedes plantarius* could presumably use Fennoscandia as a refugium, but the ability of the species to effectively spread northward has not been accounted for in previous predictions^30,31^. Moreover, fishing spiders are threatened by the decrease of range and quality of their wetland and fenland habitats, which are declining globally^33^. The other European fishing spider, *Dolomedes fimbriatus*, also occurs in Fennoscandia. Co-occurrence of both *Dolomedes*, was considered impossible due to different habitat requirements^34^. *D. fimbriatus* can nonetheless occupy the same habitat type as *D. plantarius* plus marshes, bogs, swampy forests or wet heathland^34^. Syntopy is then possible, as the two species can live close to each other^35^, for example around the same lake^36^, or in the ecotone habitat between bogs and ponds^37^. *D. fimbriatus* has a larger ecological niche: the species is more drought and shade tolerant^38^, e.g. it creates nurseries to lay eggs in the tall grass while *D. plantarius* creates nurseries only above the water surface^34^. *D. frimbriatus* is less sensitive to water quality^35^, it is found on mesotrophic or oligotrophic wetlands while *D. plantarius* lives mainly in mesotrophic wetlands^38^. Consequently, *D. fimbriatus* could become a competitor to *D. plantarius* in syntopic sites if global change brings more frequent drought events.

**Figure 1:**
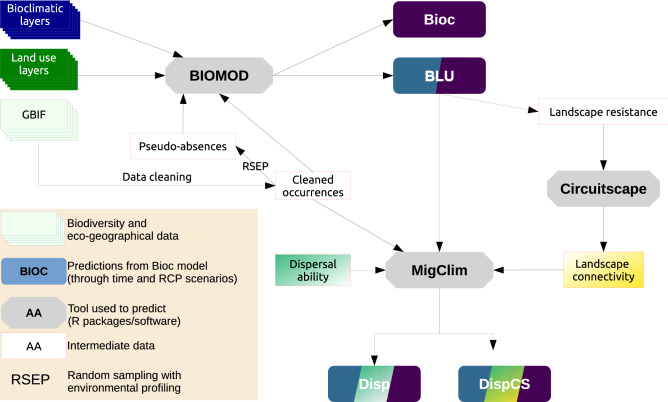
Flowchart of the framework used to study the future distribution of the two European fishing spiders (Bioc: bioclimatic only model, BLU: bioclimatic and land use model, Disp: dispersal model, DispCS: dispersal and landscape connectivity model).

Here, we compare the potential range spread of *D. plantarius* and *D. fimbriatus,* and their ability to use Fennoscandia as a refugium. We aim to provide more conservative predictions for Fennoscandia than previously predicted at the European scale by Leroy et al^30,31^. To do so, we developed hybrid species distribution models including climate and land-use variables, as well as dispersal and landscape connectivity (Fig. 1). We expected that:

1. The distribution of both fishing spiders should expand northward^30,31^. A larger expansion is expected under more intense climate change.
2. Since *D. fimbriatus* is a habitat generalist, the range of habitat it can reach should be larger and occupied faster, than for *D. plantarius*^39^.
3. The area of sympatry between the two species should increase with the range expansion of the two species.

## Material and methods

### Occurrence data

**Figure 2:**
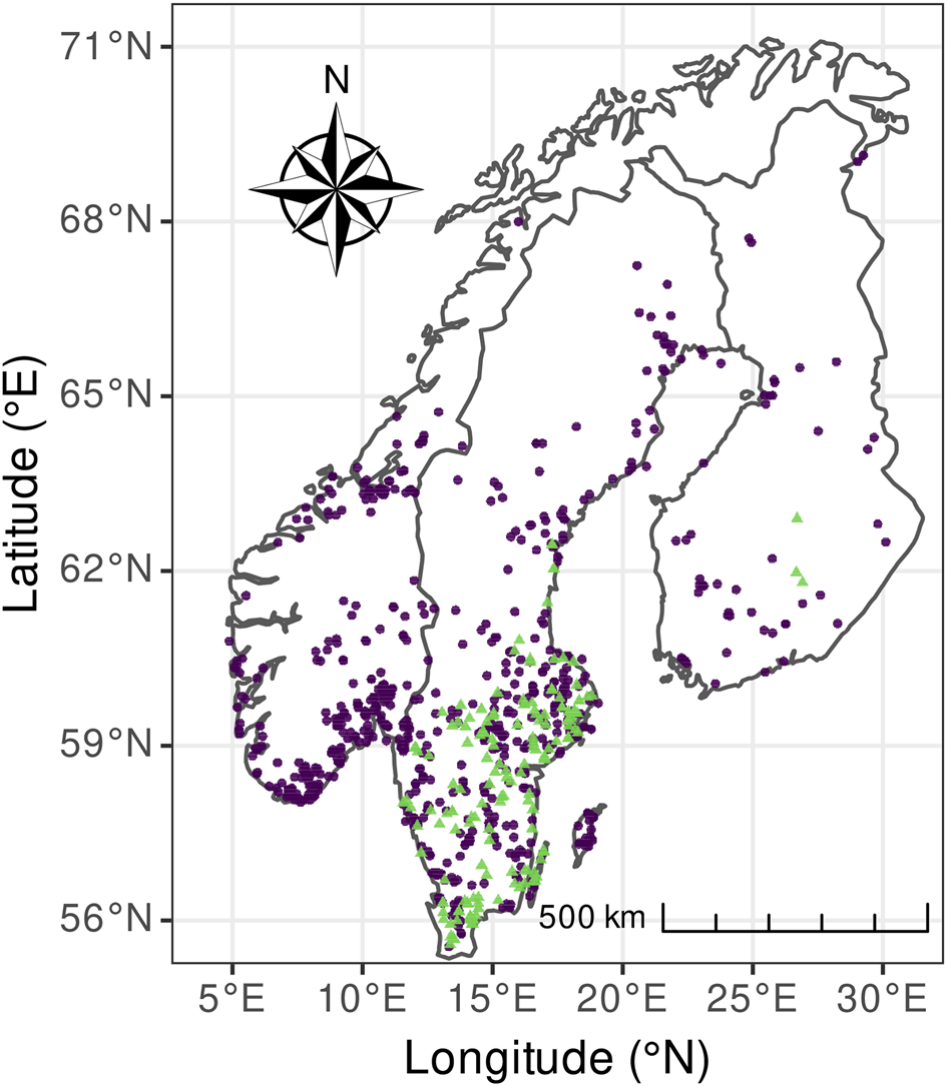
*Dolomedes plantarius* (green triangles) and *Dolomedes fimbriatus* (purple dots) records in Fennoscandia as of October 2019. Data were extracted from the GBIF database and supplemented by field samplings. The figure was created using R v.4.0.2^41^ (https://www.R-project.org/) and the R packages ggspatial v.1.1.4^102^ (https://CRAN.R-project.org/package=ggspatial), ggplot2 v.3.3.2^103^ (https://ggplot2.tidyverse.org) and rnaturalearth v.0.1.0^104^ (https://CRAN.R-project.org/package=rnaturalearth).

We downloaded records of presence for both spider species from the GBIF^40^ via the rgbif package (citations for R packages are provided in Supplementary Material 1) in R^41^. The GBIF database gathers volunteer-based naturalist observations (Supplementary Material 2), which often require a quality check. We used the package CoordinateCleaner (Supplementary Material 1) to remove null or duplicate coordinates, and to flag the records requiring a subjective decision, such as old records or records located in urban areas, or at the centroid of a county. Urban records were not necessarily false presence, and we used aerial photography^42^ accessed with packages leaflet and mapedit (Supplementary Material 1) to decide whether to keep these records or not. We visually checked, for instance, if a record was not in a recently modified areas in a city. Some records suggesting co-occurrence of the two species were checked in the field during summer 2018 and 2019 (25 locations, including four actually syntopic locations). We retained 775 records for *Dolomedes fimbriatus* and 181 records for *Dolomedes plantarius* (Fig. 2), reflecting the GBIF data available until October 2019 in Fennoscandia. When several records fell in the same raster cell, we kept only one.

### Species distribution modelling

#### Predictor variables

For the climatic component of the ecological niche, we included variables which were biologically relevant for spiders, and not too correlated^43^. Using a correlation coefficient threshold of 0.7^44^, we selected mean and maximum annual temperature, mean diurnal temperature range, mean temperature of the wettest quarter, and annual precipitation, which we extracted from the WorldClim database^45^ at a spatial resolution of 30 arc-seconds (Supplementary Material Table S2).

To predict the future distribution of *Dolomedes* spiders in Fennoscandia, we used IPCC projections for 2050 and 2070, under multi-factors “representative concentration pathways” (RCP) 4.5 and 8.5^46^. RCP4.5 corresponds to medium–low greenhouse gas emissions and air pollution, whereas RCP8.5 considers high greenhouse gas emission, medium air pollution, and an increase in carbon dioxide^46^. We downloaded these climatic projections from Wordclim^47^ at a spatial resolution of 30 arc-sec.

For the habitat component of the ecological niche, we integrated information on ground wetness, which is an important community driver for the semi-aquatic fishing spiders^29,48^. We also incorporated forest and grassland density, because the presence of fishing spiders seems to be influenced by the surrounding landscape^49^. We downloaded the corresponding geographic layers from the Copernicus Land Monitoring Service at 100-m resolution^50^, and upscaled them to 30 arc-seconds resolution to match the bioclimatic data. The forest layer represents the density of the tree cover (from 0 to 100%) in 2015. The ‘Water and Wetness’ layer represents the occurrence of wet surfaces from 2009 to 2015, using a water and wetness probability index, indicating the degree of physical wetness, independently of the vegetation cover. Finally, the grassland layer represents the percentage of grassland per pixel. We estimated the change in land use between current and future times with a model which harmonises scenarios from different integrated assessment models, namely MESSAGE for RCP8.5 and GCAM for RCP4.5^51^.

#### Calibration area and pseudo-absences

To use presence-absence models with the presence-only GBIF data, we used a random sampling procedure with environmental profiling^52^. Which creates a background of absence records for each algorithm. We generated the pseudo-absences in a different calibration area for each species. *D. plantarius* is a lowland species, so its calibration area was at low altitude < 1000 m. For *D. fimbriatus*, we excluded areas > 1500 m.

#### Model validation

Although there are many SDMs, none stands out as better than the others^14^. To improve the predictions, we therefore used an ensemble forecast approach, which combines several models weighted by their predictive accuracy^53,54^.

Following recommendations in Barbet-Massin et al.^55^, we built our ensemble model with 10 runs of gradient boosting models (GBMs), generalized additive models (GAMs) and Maxent. We used 1000 pseudo-absences for the GBMs, and as many pseudo-absences as presences for the GAMs. We used 80% of the data for training the ensemble model and testing the single run of model, and 20% for validation. Each model was cross-validated with a fivefold procedure in package biomod2 (Supplementary Material 1), thus leading to 5 fits for each type of model and each pseudo-absences run. We then evaluated the predictive accuracy of individual models with the true skill statistic (TSS) and the area under the receiving operating curve (AUROC). The TSS metric represents the ratio of hit rate to false alarm rate and varies from − 1 to + 1^56^. We used a threshold of TSS = 0.4 to include models into the ensemble forecast^56^. The AUROC is a measure of "separability", which represents the true positive rates graphically against the true negative rates. Following Fawcett^57^, we retained models with AUC > 0.7 for the ensemble model. Finally, we converted the probabilities of presence predicted by the ensemble model into a binary presence/absence, with a cut point based on predictions which maximized the TSS (Supplementary Material 1). In package biomod2, the relative variable contribution is assessed based on the correlation between the prediction of a model including a given variable and the model where this variable was dropped.

We built one model with bioclimatic variables only (model Bioc), and one with bioclimatic and land-use variables (model BLU). We then included dispersal to predict the range of suitable, but unreachable habitat (model Disp). Finally, we accounted for landscape connectivity into model dispCS. The framework is summarized in Fig. 1 (additional details in Supplementary Material Table S3).

### Including dispersal into SDM

Although they differ in their general dispersal ability, the two species of fishing spider disperse mostly through ballooning and rappelling, where they catch the wind with a thread of silk, and passively fly. Laboratory tests suggested that few individuals exhibit long-distance dispersal behaviour on the water surface (unpublished data). We recorded this behaviour only in *Dolomedes fimbriatus* through sailing (when spider raised its body and/or abdomen and/or the legs to catch the wind). However, juveniles of *D. fimbriatus* are generally found in the surrounding vegetation rather than on the water^35^, which makes aquatic dispersal unlikely.

We modelled dispersal ability via the MigClim package (Supplementary Material 1), based on the predicted map of the BLU model. For each species, the MigClim model evaluates if suitable cells of the raster could become accessible between current time and 2050/2070. The package uses a dispersal kernel, i.e., a vector of probabilities of dispersal, to simulate the dispersal of the species (Supplementary Table S1). We used an imperviousness map50 to locate areas where the species settlement is highly unlikely. Since both fishing spiders are water-dependent, impervious regions where the soil seals, are barrier to settlement. Part of the MigClim modelling process is random^58^, so we replicated each model 30 times and model-averaged the estimates.

In experimental settings, aerial dispersal (ballooning) is usually characterized when the spider is observed tiptoeing in response to a controlled wind. However, not all tiptoeing spiders end up ballooning^59,60^. The distance covered by aerial dispersal is less than 5 km on average and is not correlated with the duration of the tiptoeing behaviour^61^. We parametrized the MigClim model with values from the literature on aerial dispersal distance in spiders^61,62^. We weighed these values by the proportion of individuals we observed rappelling in our laboratory experiments (Monsimet et al. in prep), namely, 76.6% of *D. fimbriatus* and 59% *D. plantarius*. For long-distance dispersal, we used the proportion of individuals observed ballooning (*D. fimbriatus*: 14%, *D. plantarius*: 2.9%) for 2019. We considered that the probability of a settlement was similar for both species. Also, we hypothesized that it takes two years for a newly colonized area to produce new propagules, based on the > 2-year lifespan of spiders in Northern Europe^35^.

### Accounting for landscape connectivity

We used the Circuitscape software^63^ to predict the potential dispersal corridors that *Dolomedes* could use to colonize their suitable habitat. Circuit theory estimates multiple pathways based on the resistance and conductance of the landscape^64^. We used the habitat suitability prediction map from our BLU model to define the resistance map used by Circuitscape. We transformed the estimates of habitat suitability according to recommendations in Keeley^65^ (see also Supplementary Material 3).

We used a "wall-to-wall" approach^66,67^ which estimates the conductivity of the landscape from South to North, and from West to East. A consensus map was produced by multiplying the resistance layers of different directions. This consensus map was an estimation of the landscape connectivity for the two species. The consensus map was binarized by considering conductance higher than mean conductance plus standard deviation as corridors^67^. Areas outside corridors were then considered as a barrier to short-distance dispersal in Migclim. Migclim was parametrized as for the model Disp but accounting for the landscape connectivity barrier to make predictions for model DispCS.

### Range expansion and geographic overlap in time

We compared suitable habitat predicted across species, models, and scenarios. To estimate the range expansion or reduction in the future, we used the biomod2 package in R. We compared the direction of the shift in suitable habitat by calculating the centre of gravity of the suitable range with the SDMTools package (Supplementary Material 1). To estimate the overlap of suitable habitat range between species for each time/scenario combination, we used the Schoeners’ D overlap metric^68^, which ranges from 0 for no overlap to 1 for full overlap^69^. We estimated the suitable habitat range overlap and not the full niche overlap here. We calculated D with the ENMtools package (Supplementary Material 1).

## Results

### Modelling and model validation

The predictive performance of both Bioc and BLU models was higher than the threshold with either the ROC (> 0.7) or the TSS (> 0.4) metric (Supplementary Material Table S3). The relative contribution of predictors was the same across models and species, with mean annual temperature the most important variable with a contribution higher than 60%. For Bioc, mean temperature of the warmest month was also important, with a higher contribution for *D. fimbriatus* than for *D. plantarius* (33% and 11%, respectively). Mean temperature of the wettest quarter, annual precipitation and mean diurnal range contributed less than 10% to both models. Forest and ground wetness contributed more than grassland in the BLU models, but their relative contribution was less than 16%.

### Range expansion and geographic overlap in time

**Figure 3:**
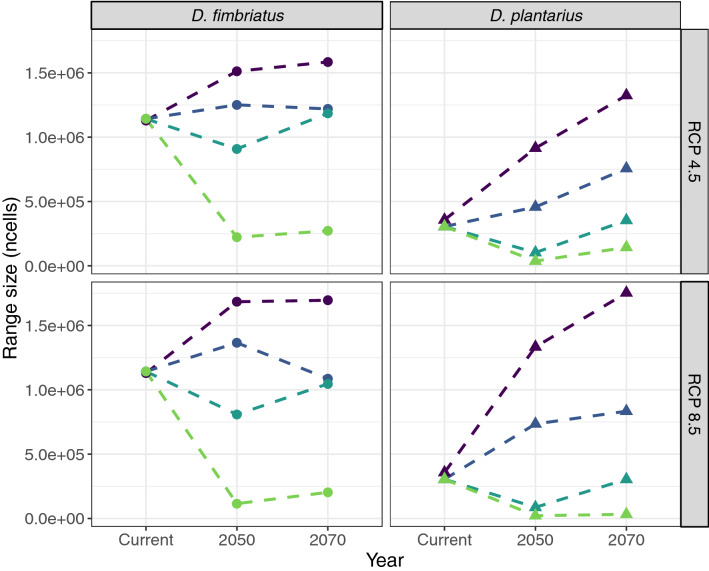
Range size in number of cells of suitable habitat predicted by the different SDMs in time per species and scenarios (dark purple: Bioc model: bioclimatic variables only; dark blue: BLU model, bioclimatic + land use; turquoise: Disp model with dispersal; green: DispCS model: dispersal and landscape connectivity). The figure was created using R v.4.0.2^41^ (https://www.R-project.org/) and the R package ggplot2 v.3.3.2^103^ (https://ggplot2.tidyverse.org).

The size of the predicted/projected range was similar for both Bioc and BLU models. However, range expansion was predicted to be more restricted when also accounting for land use (BLU) than when considering only climatic variables (Bioc). Indeed, adding land use variables contracted the suitable habitat at the limit of the range. Suitable range was also smaller for RCP4.5 than for RCP8.5, with similar patterns in time, except for *D. fimbriatus* where the range was reduced in 2070 compared to current under model BLU (Fig. 3; Supplementary Material Table S4).

**Figure 4:**
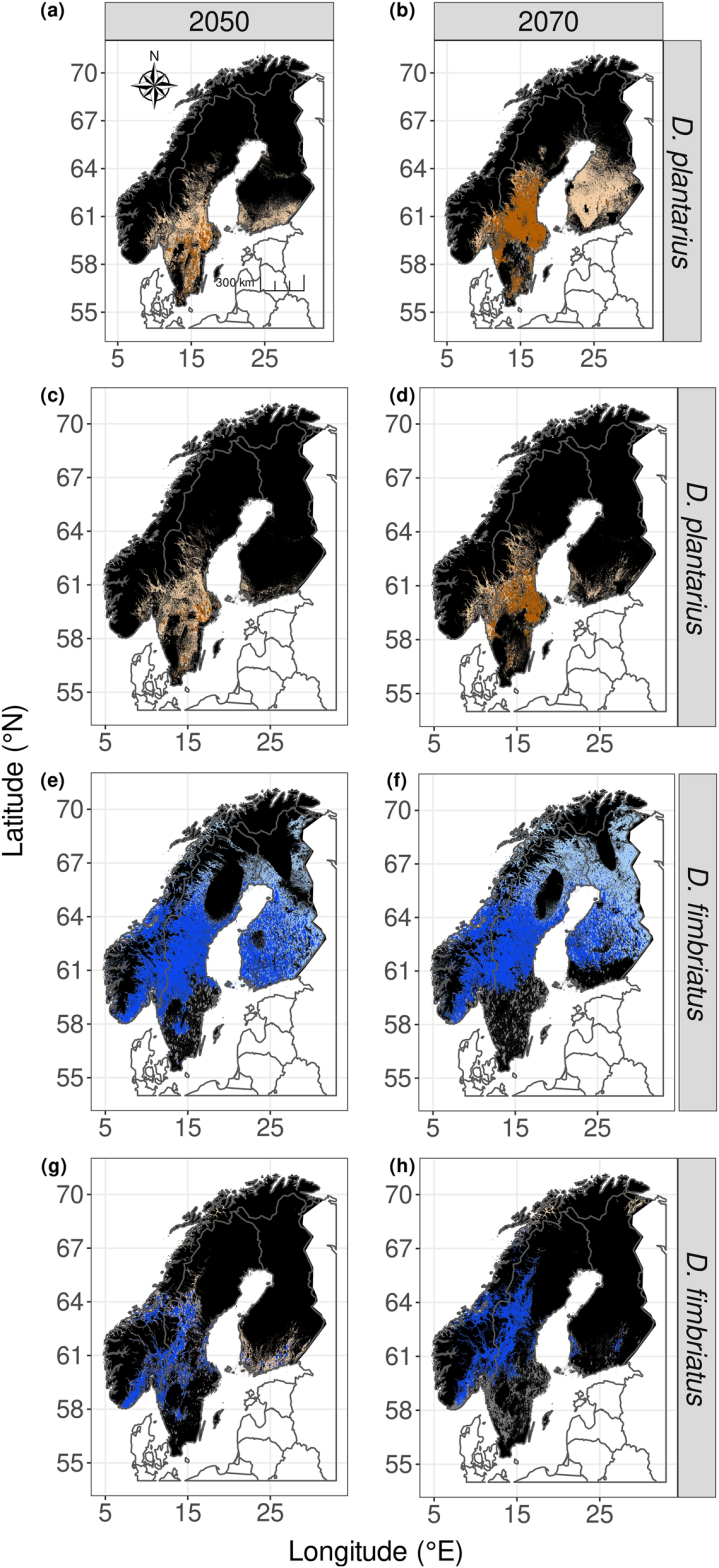
Map of the forecasted suitable habitat with an estimation of the reachable range predicted by the dispersion model (Disp) and reachable area from the connectivity model (DispCS) under the RCP4.5 scenario (RCP: representative concentration pathway; in dark brown the reachable habitat for D. plantarius under Disp (**a** and **b**) and DispCS (**c** and **d**); in dark blue the reachable for D. fimbriatus under Disp (**e** and **f**) and DispCS (**g** and **h**); in black: unsuitable habitat; in grey: previously occupied habitat lost; in light brown and light blue: suitable but non reachable habitat). The figure was created using R v.4.0.2^41^ (https://www.R-project.org/) and the R packages ggspatial v.1.1.4^102^ (https://CRAN.R-project.org/package=ggspatial), ggplot2 v.3.3.2^103^ (https://ggplot2.tidyverse.org), rnaturalearth v.0.1.0^104^ (https://CRAN.R-project.org/package=rnaturalearth) and ggpubr v.0.4.0^105^ (https://CRAN.R-project.org/package=ggpubr).

Under RCP4.5 scenario, the suitable range was predicted to increase for both species in 2070 with the BLU model (14% for *D. fimbriatus* and 161% for *D. plantarius*). With model Disp, the range should decrease in 2050 for *D. fimbriatus* (20% decrease) and for *D. plantarius* (66% decrease; Fig. 3). Both species should be able to occupy the suitable range towards 2070, but both should have a limited range expansion of suitable habitat under Disp (Figs. 3 and 4; 14% increase under BLU and 4% under Disp for *D. fimbriatus*; 161% and 16%, respectively, for *D. plantarius*). The range of both species should shrink under DispCS (81% in 2050 and 76% in 2070, compared to current suitable habitat for *D. fimbriatus*; 88% and 53%, respectively, for *D. plantarius*).

**Figure 5:**
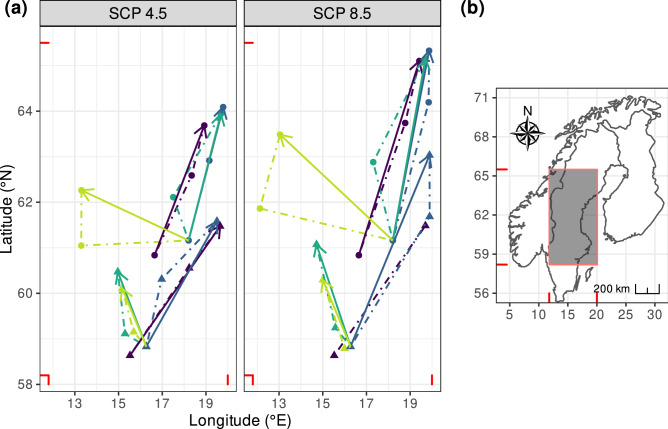
Shift in the centre of gravity of the two species distributions predicted by the four SDMs; solid lines: shift from current to 2070; dashed lines: shift from current time to 2050 and from 2050 to 2070. Dark purple: Bioc model; dark blue: BLU model; turquoise: Disp model; green: DispCS model. The figure was created using R v.4.0.2^41^ (https://www.R-project.org/) and the R packages ggspatial v.1.1.4^102^ (https://CRAN.R-project.org/package=ggspatial), ggplot2 v.3.3.2^103^ (https://ggplot2.tidyverse.org), rnaturalearth v.0.10^104^ (https://CRAN.R-project.org/package=rnaturalearth) and ggpubr v.0.4.0^105^ (https://CRAN.R-project.org/package=ggpubr).

The southern part of the suitable range should shrink, especially in Sweden and, to a lesser extent, in Finland. This range should expand in northern Fennoscandia (Fig. 4). According to model dispCS, this shift should occur towards the North-East, with a limited spread in southern Finland (Fig. 3). Similarly, the range of suitable habitat for *D. plantarius* should also increase towards the North-East under model Disp (Fig. 5). The shift of the centre of gravity is at a higher distance for the models which exclude Dispersal (Bioc and BLU) than model including dispersal (Disp and DispCS). The centre of gravity shifts farther without dispersal (models Bioc and BLU) than with dispersal (models Disp and DispCS).

**Figure 6:**
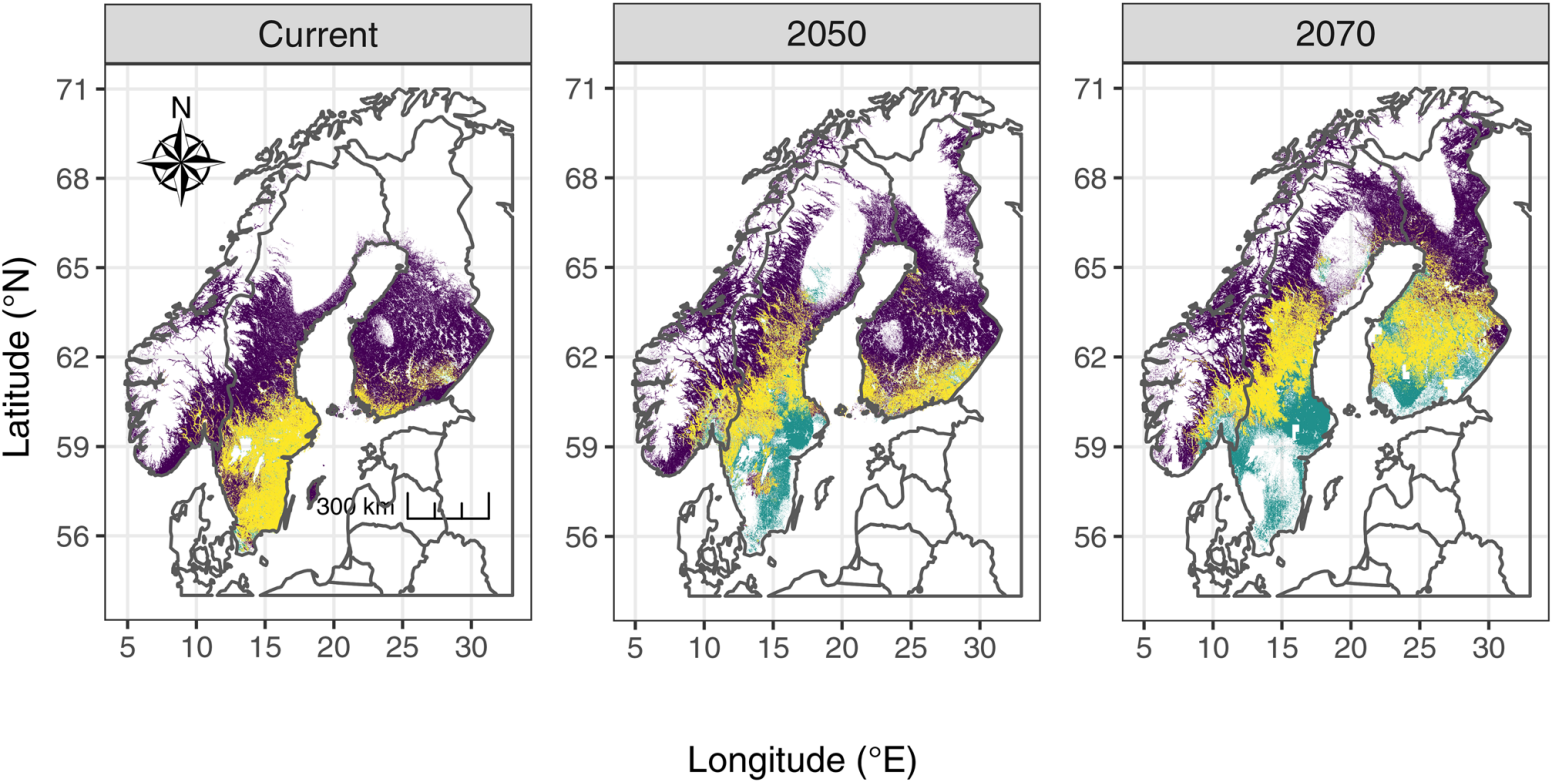
Range overlap predicted by model BLU from current time to 2070 under scenario RCP4.5. In addition to overlap of suitable range, suitable habitat for each species is represented. Dark purple: suitable habitat for *D. fimbriatus*; green: suitable habitat for *D. plantarius*; yellow: suitable habitat overlapping between the 2 species. The figure was created using R v.4.0.2^41^ (https://www.R-project.org/) and the R packages ggspatial v.1.1.4^102^ (https://CRAN.R-project.org/package=ggspatial), ggplot2 v.3.3.2^103^ (https://ggplot2.tidyverse.org), rnaturalearth v.0.10^104^ (https://CRAN.R-project.org/package=rnaturalearth) and ggpubr v.0.4.0^105^ (https://CRAN.R-project.org/package=ggpubr).

The predicted distribution overlap between species was higher when considering only climatic variables than when accounting for land use at current time (Bioc model). Under the BLU model, the overlap should increase through time and is more important for the scenario SRCRCP8.5 than the 4.5 one (Schoener’s D values ranging from 0.55 at current time to 0.62 in 2070 for RCP4.5, it reached 0.68 under 8.5). The overlap should mainly occur at the Southern range of *Dolomedes fimbriatus* distribution (Fig. 6; Supplementary Material Table S5).

## Discussion

Using species distribution models (SDMs), we highlighted different range expansions and shifts of two closely related fishing spiders species in Fennoscandia. According to our predictions, the range of suitable habitat should expand for both *D. fimbriatus* and *D. plantarius.* Our climatic and habitat models (Bioc and BLU) confirmed the expansion of *D. plantarius* in Fennoscandia predicted by Leroy et al.^30,31^. In contrast, our hybrid models including dispersal and landscape connectivity (Disp and DispCS) predicted a more limited expansion*.*

### Northward range expansion of both *Dolomedes* species

A northward expansion in Fennoscandia is expected for the two species under both Bioc and BLU models. The range of suitable habitat should increase with the intensity of the climate change for *D. plantarius* and for *D. fimbriatus* in 2050. This northward expansion is also predicted in other taxa, as climate change promote an expansion of the range at the colder margin^4,5^. An increase in annual mean temperature and in temperature of the warmest month, which are the most important variables for both models, could impact the lifespan of the two spider species, and affect their distribution. Higher temperatures could increase the suitable period to produce juveniles, which could in turn increase the number of juveniles dispersing. The temperature encountered by juveniles also influences the dispersal ability and mode (i.e., long vs short distance dispersal^70^). Moreover, latitude and climate affect the time at which the *Dolomedes* reach maturity^35^. This could increase the frequency of a second brood, which we already observed in September (unpublished data). Such an increase in temperature could, in turn, influence the speed of colonization of new habitats. The inclusion of land use in BLU models shrinks the range of suitable habitat, which confirms results from other, similar studies^71^.

Under the Disp model, suitable habitat should be less reachable for *D. plantarius* than for *D. fimbriatus.* The size of the area reached under the Disp model should be smaller than the current area for both species. In 2070, *D. fimbriatus* should have a range slightly equivalent to the suitable habitat estimated under BLU, whereas it should be smaller for *D. plantarius.* The limited expansion o*f D. plantarius* is explained mainly by dispersal ability. Indeed, we observe fewer spiderlings of *D. plantarius* showing dispersal behaviours, including long-distance dispersal through ballooning (unpublished data). Differences in predicted suitable habitat and occupied habitat can be explained by either or both past and current limited dispersal, as exemplified by tree species^72^. Some species may be limited in their geographical range and their distributions may have not changed since the last glaciation. Species that either cannot or do not shift range may be responding to climate change in situ whether through microevolution or adaptive phenotypic plasticity^73^. Some species are not yet able to adjust their phenology and physiology to changes induced by climate change. The importance of short-distance dispersal in fishing spiders should nonetheless maintain genetic exchange, or avoid genetic drift, at a smaller scale^74^. A possible prevalence of this behaviour might also reinforce the importance of shorter dispersal as climate change and other factors like the increase of habitat fragmentation decrease long-distance dispersal of spiders^75^.

### Geographic range overlap and coexistence

The geographic and climatic niche of *D. plantarius* are included in the realised niche of *D. fimbriatus*. The first is a habitat specialist, the last is a more generalist species living in a wider variety of environmental conditions over its range. Climate change increases the chance of overlap between these two sister species. However, we did not make predictions at a meso- or microhabitat scale, which would be too fine for SDMs. Yet, field observations suggest that both *Dolomedes* species also co-occur at finer spatial scales^35^. The discrete nature and propensity to hide and dive of *D. plantarius*^34^, together with possible misidentification^36,76^ might explain the small number of records and of co-occurrences. In North America, closely related species of *Dolomedes* like *D. trition* and *D. vittatus* were reported to co-occur at small spatial scales^77^.

Usually, closely related species co-occur less often than moderately related species^78^. On one hand, an increase in co-occurrence might limit the distribution by segregation at the landscape scale. Indeed, the number of interactions between species in the ecosystem can increase with climate change^79^, which may result in a spatial separation between generalist and specialist species^80,81^. Sympatric sister species usually diverge ecologically^82^, *Dolomedes* species differ in terms of habitat use^35^. *D. plantarius* needs open habitat with slow-flowing water and water all year, while these factors do not seem to restrict *D. fimbriatus* (unpublished data). On the other hand, spatial segregation might occur at the micro-habitat scale. For instance, a study on *Tetragnatha* spiders showed that one of two co-existing spider species builds nursery webs higher in the vegetation when they co-occur^83^. Finally, an increase in co-occurrence might lead to phenological shift in co-existence sites. Our observation in two Swedish locations of *D. fimbriatus* females with juveniles in the nursery while *D. plantarius* still carried egg sacs could support this. Other closely related wolf spider species (Lycosidae) also show differences in the timing of their breeding season to avoid intraguild predation^84^.

### Intrinsic limits of hybrid SDMs

Ideally, a mechanistic model should account for all phases of dispersal, ie, emigration, transfer, settlement^85,86^. The SDM accounting for dispersal which we used here it not a mechanistic model but is rather based on assumptions concerning the three stages of passive dispersal. Further studies should consider factors which influence individuals’ dispersal such as food availability^87^, presence of endosymbionts^88^, presence of conspecific in the short-long distance dispersal allocation^89^, or genetically inherited propensity for dispersal via ballooning^90^. Since dispersal is not homogeneous within and among species^91^, a more realistic model should include information on dispersal and population size for each presence observation. The sampling of all sites is necessary to collect this information. There is a considerable gap between the theory and actual applications of data-demanding mechanistic SDMs^26^. Knowing that the most used habitat is not necessarily the most suitable for the fitness of the species^92^, we used a hybrid model based on the lack of sufficient data for a full mechanistic model.

Moreover, accounting for thermal niche information is possible with mechanistic models^93,94^. Including the lower lethal limit of *Dolomedes* could be relevant to estimate their future distributions. Indeed, we used air temperature data to characterize the temperature in our SDMs, but *Dolomedes* spiders overwinter under the snow. Climate change is impacting the snow cover, and thus, the insulation of the subnivean habitat, which is getting colder^95^. However, the current knowledge of eco-physiological responses of fishing spiders to climate change is too scarce to allow fully mechanistic models.

### Conservation of fishing spiders

Fennoscandia may become a climatic refugium for *D. plantarius* as its range in continental Europe is expected to decrease^30,31^. The more extreme the climate change is, the more likely Fennoscandia will act as a refugium. The overlap between the two *Dolomedes* species should also increase with the climate change intensity. Arthropod conservation is challenging because of the fine-grain level needed as compared to vertebrates, the low empathy towards invertebrates, and the lowest number of conservation specialists available^96,97^. Nonetheless, spiders have already been used as bio-indicators^98,99^. Our models suggest that the conservation of both species is necessary as the reachable range size should drastically decrease in the future when accounting for dispersal and landscape connectivity. Conservation of preserved sites in a stepping-stones scheme is an alternative for species that are not able to use corridors^100^. Maintaining interconnected suitable sites in the first five kilometres around sites with known presence should help conserve current sites and promote expansion. With respect to fishing spiders, priority should be given to sites in southern Finland and central Sweden, where there is limited connectivity, and the spread of *Dolomedes* species is limited. Since *D. fimbriatus* has higher dispersal abilities, improving the connectivity in the North of the suitable range to make it reachable should improve the future range.

This work, together with other studies on *Dolomedes*, could be used to update the now outdated range assessment of *D. plantarius*^101^. The species’ conservation would benefit from such an update.

## Supplementary information

Supplementary Information.

## Data Availability

The datasets generated during and/or analyzed during the current study and interactive maps of the predictions of suitable/reachable habitats are available (10.18710/TYPJXU).

## References

[1] Bellard C, Bertelsmeier C, Leadley P, Thuiller W, Courchamp F (2012). Impacts of climate change on the future of biodiversity: biodiversity and climate change. Ecol. Lett..

[2] Garcia RA, Cabeza M, Rahbek C, Araújo MB (2014). Multiple dimensions of climate change and their implications for biodiversity. Science.

[3] Pereira HM (2010). Scenarios for global biodiversity in the 21st century. Science.

[4] Parmesan C, Yohe G (2003). A globally coherent fingerprint of climate change impacts across natural systems. Nature.

[5] Parmesan C (2006). Ecological and evolutionary responses to recent climate change. Annu. Rev. Ecol. Evol. Syst..

[6] Walther G-R (2002). Ecological responses to recent climate change. Nature.

[7] Thomas CD (2004). Extinction risk from climate change. Nature.

[8] Miller J (2010). Species distribution modeling. Geogr, Compass.

[9] Guisan A (2013). Predicting species distributions for conservation decisions. Ecol. Lett..

[10] Bellard C (2013). Will climate change promote future invasions?. Glob. Change Biol..

[11] Hijmans RJ, Graham CH (2006). The ability of climate envelope models to predict the effect of climate change on species distributions. Glob. Change Biol..

[12] Hao T, Elith J, Guillera-Arroita G, Lahoz-Monfort JJ (2019). A review of evidence about use and performance of species distribution modelling ensembles like BIOMOD. Divers. Distrib..

[13] Melo-Merino SM, Reyes-Bonilla H, Lira-Noriega A (2020). Ecological niche models and species distribution models in marine environments: a literature review and spatial analysis of evidence. Ecol. Model..

[14] Qiao H, Soberón J, Peterson AT (2015). No silver bullets in correlative ecological niche modelling: insights from testing among many potential algorithms for niche estimation. Methods Ecol. Evol..

[15] Araújo MB, New M (2007). Ensemble forecasting of species distributions. Trends Ecol. Evol..

[16] Thuiller W (2004). Patterns and uncertainties of species’ range shifts under climate change. Glob. Change Biol..

[17] Thuiller W, Guéguen M, Renaud J, Karger DN, Zimmermann NE (2019). Uncertainty in ensembles of global biodiversity scenarios. Nat. Commun..

[18] Titeux N (2016). Biodiversity scenarios neglect future land-use changes. Glob. Change Biol..

[19] Solomon, S. *et al.**IPCC, 2007: Climate Change 2007: The Physical Science Basis. Contribution of Working Group I to the Fourth Assessment Report of the Intergovernmental Panel on Climate Change*, Vol. 1 (2007).

[20] Guisan A, Thuiller W (2005). Predicting species distribution: offering more than simple habitat models. Ecol. Lett..

[21] Richmond OMW, McEntee JP, Hijmans RJ, Brashares JS (2010). Is the climate right for pleistocene rewilding? Using species distribution models to extrapolate climatic suitability for mammals across continents. PLoS ONE.

[22] Kearney M (2006). Habitat, environment and niche: what are we modelling?. Oikos.

[23] Soberon J, Peterson AT (2005). Interpretation of models of fundamental ecological niches and species’ distributional areas. Biodivers. Inform..

[24] Merow C, LaFleur N, Silander JA, Wilson AM, Rubega M (2011). Developing dynamic mechanistic species distribution models: predicting bird-mediated spread of invasive plants across northeastern North America. Am. Nat..

[25] Bocedi G (2014). RangeShifter: a platform for modelling spatial eco-evolutionary dynamics and species’ responses to environmental changes. Methods Ecol. Evol..

[26] Briscoe NJ (2019). Forecasting species range dynamics with process-explicit models: matching methods to applications. Ecol. Lett..

[27] Thuiller W, Lafourcade B, Engler R, Araújo MB (2009). BIOMOD—a platform for ensemble forecasting of species distributions. Ecography.

[28] Mammola S, Isaia M (2017). Rapid poleward distributional shifts in the European cave-dwelling Meta spiders under the influence of competition dynamics. J. Biogeogr..

[29] Lafage D, Maugenest S, Bouzillé J-B, Pétillon J (2015). Disentangling the influence of local and landscape factors on alpha and beta diversities: opposite response of plants and ground-dwelling arthropods in wet meadows. Ecol. Res..

[30] Leroy B (2013). First assessment of effects of global change on threatened spiders: potential impacts on *Dolomedes Plantarius* (Clerck) and its conservation plans. Biol. Conserv..

[31] Leroy B (2014). Forecasted climate and land use changes, and protected areas: the contrasting case of spiders. Divers. Distrib..

[32] Keppel G, Wardell-Johnson GW (2012). Refugia: keys to climate change management. Glob. Change Biol..

[33] Finlayson CM (2019). The second warning to humanity—providing a context for wetland management and policy. Wetlands.

[34] van Helsdingen PJ (1993). Ecology and distribution of dolomedes in Europe (Araneida: Dolomedidae). Boll. Acc. Gioenia Sci. Nat..

[35] Duffey E (2012). *Dolomedes plantarius* (Clerck, 1757) (Araneae: Pisauridae): a reassessment of its ecology and distribution in Europe, with comments on its history at Redgrave and Lopham Fen, England. Bull. Br. Arachnol. Soc..

[36] Ivanov V, Prishepchik O, Setrakova E (2017). *Dolomedes plantarius* (Araneae, Pisauridae) in Belarus: records, distribution and implications for conservation. Arachnol. Mitteilungen.

[37] Holec, M. Spiders (aranea) of the fishpond eulittoral zone. In *Proceedings of the 18th European Colloquium of Arachnology* vol. 19, 51–54 (Ekológia, Bratislava, 2000).

[38] Duffey, E. The distribution, status and habitat of *Dolomedes fimbriatus* (Clerck) and *D. plantarius* (Clerck) in Europe. In *Proceedings of 15th European Colloquium of Arachnology* 54–65 (1995).

[39] Hill JK, Thomas CD, Blakeley DS (1999). Evolution of flight morphology in a butterfly that has recently expanded its geographic range. Oecologia.

[40] *GBIF: The Global Biodiversity Information Facility. What is GBIF?* https://www.gbif.org/what-is-gbif (2019).

[41] R Core Team. R: A Language and Environment for Statistical Computing*. R Foundation for Statistical Computing, Vienna, Austria*(2020).

[42] ESRI. World Imagery. (2009).

[43] Braunisch V (2013). Selecting from correlated climate variables: a major source of uncertainty for predicting species distributions under climate change. Ecography.

[44] Dormann CF (2007). Promising the future? Global change projections of species distributions. Basic Appl. Ecol..

[45] Fick SE, Hijmans RJ (2017). WorldClim 2: new 1-km spatial resolution climate surfaces for global land areas. Int. J. Climatol..

[46] van Vuuren DP (2011). The representative concentration pathways: an overview. Clim. Change.

[47] Hijmans RJ, Cameron SE, Parra JL, Jarvis A (2005). Very high resolution interpolated climated surfaces for global land areas. Int. J. Climatol..

[48] Lafage D, Pétillon J (2016). Relative importance of management and natural flooding on spider, carabid and plant assemblages in extensively used grasslands along the Loire. Basic Appl. Ecol..

[49] Dickel, L. *Characterisation of Habitat Requirements of European Fishing Spiders* (Inland Norway University of Applied Sciences, 2019).

[50] EEA. European Union, Copernicus Land Monitoring Service 2018, European Environment Agency (EEA). (2018).

[51] Hurtt GC (2011). Harmonization of land-use scenarios for the period 1500–2100: 600 years of global gridded annual land-use transitions, wood harvest, and resulting secondary lands. Clim. Change.

[52] Senay SD, Worner SP, Ikeda T (2013). Novel three-step pseudo-absence selection technique for improved species distribution modelling. PLoS ONE.

[53] Grenouillet G, Buisson L, Casajus N, Lek S (2011). Ensemble modelling of species distribution: the effects of geographical and environmental ranges. Ecography.

[54] Buisson L, Thuiller W, Casajus N, Lek S, Grenouillet G (2010). Uncertainty in ensemble forecasting of species distribution. Glob. Change Biol..

[55] Barbet-Massin M, Jiguet F, Albert CH, Thuiller W (2012). Selecting pseudo-absences for species distribution models: how, where and how many?. Methods Ecol. Evol..

[56] Allouche O, Tsoar A, Kadmon R (2006). Assessing the accuracy of species distribution models: prevalence, kappa and the true skill statistic (TSS). J. Appl. Ecol..

[57] Fawcett T (2006). An introduction to ROC analysis. Pattern Recognit. Lett..

[58] Engler R, Guisan A (2009). MigClim: predicting plant distribution and dispersal in a changing climate. Divers. Distrib..

[59] Bonte D, Clercq ND, Zwertvaegher I, Lens L (2009). Repeatability of dispersal behaviour in a common dwarf spider: evidence for different mechanisms behind short- and long-distance dispersal. Ecol. Entomol..

[60] Lee VMJ, Kuntner M, Li D (2015). Ballooning behavior in the golden orbweb spider Nephila pilipes (Araneae: Nephilidae). Front. Ecol. Evol..

[61] Reynolds AM, Bohan DA, Bell JR (2007). Ballooning dispersal in arthropod taxa: conditions at take-off. Biol. Lett..

[62] Thomas CFG, Brain P, Jepson PC (2003). Aerial activity of linyphiid spiders: modelling dispersal distances from meteorology and behaviour. J. Appl. Ecol..

[63] Shah, V. B. & McRae, B. Circuitscape: a tool for landscape ecology. In *Proceedings of the 7th Python in Science Conference* Vol. 7 62–66 (2008).

[64] McRae BH, Dickson BG, Keitt TH, Shah VB (2008). Using circuit theory to model connectivity in ecology, evolution, and conservation. Ecology.

[65] Keeley ATH, Beier P, Keeley BW, Fagan ME (2017). Habitat suitability is a poor proxy for landscape connectivity during dispersal and mating movements. Landsc. Urban Plan..

[66] Pelletier D (2014). Applying circuit theory for corridor expansion and management at regional scales: tiling, pinch points, and omnidirectional connectivity. PLoS ONE.

[67] Febbraro MD (2019). Integrating climate and land-use change scenarios in modelling the future spread of invasive squirrels in Italy. Divers. Distrib..

[68] Warren DL, Glor RE, Turelli M (2008). Environmental niche equivalency versus conservatism: quantitative approaches to niche evolution. Evol. Int. J. Org. Evol..

[69] Rödder D, Engler JO (2011). Quantitative metrics of overlaps in Grinnellian niches: advances and possible drawbacks. Glob. Ecol. Biogeogr..

[70] Bonte D, Travis JMJ, Clercq ND, Zwertvaegher I, Lens L (2008). Thermal conditions during juvenile development affect adult dispersal in a spider. Proc. Natl. Acad. Sci..

[71] Eskildsen A (2013). Testing species distribution models across space and time: high latitude butterflies and recent warming. Glob. Ecol. Biogeogr..

[72] Svenning J-C, Skov F (2004). Limited filling of the potential range in European tree species. Ecol. Lett..

[73] Radchuk V (2019). Adaptive responses of animals to climate change are most likely insufficient. Nat. Commun..

[74] Bell JR, Bohan DA, Shaw EM, Weyman GS (2005). Ballooning dispersal using silk: world fauna, phylogenies, genetics and models. Bull. Entomol. Res..

[75] Bonte D, Borre JV, Lens L, Jean-Pierre M (2006). Geographical variation in wolf spider dispersal behaviour is related to landscape structure. Anim. Behav..

[76] Bellvert A, Casals A, Fonollosa A, Dalmau G, Tobella C (2013). First record of *Dolomedes plantarius* (Clerck, 1758) (Araneae: Pisauridae) from the Iberian Peninsula. Rev. Ibérica Aracnol..

[77] Carico JE (1973). The nearctic species of the genus Dolomedes (Araneae: Pisauridae). Bull. Mus. Comp. Zool. Harv. Coll..

[78] Weinstein BG, Graham CH, Parra JL (2017). The role of environment, dispersal and competition in explaining reduced co-occurrence among related species. PLoS ONE.

[79] Montoya JM, Raffaelli D (2010). Climate change, biotic interactions and ecosystem services. Philos. Trans. R. Soc. B Biol. Sci..

[80] Warren MS (2001). Rapid responses of British butterflies to opposing forces of climate and habitat change. Nature.

[81] Roux PCL, McGeoch MA (2008). Rapid range expansion and community reorganization in response to warming. Glob. Change Biol..

[82] Losos JB (2008). Phylogenetic niche conservatism, phylogenetic signal and the relationship between phylogenetic relatedness and ecological similarity among species. Ecol. Lett..

[83] Williams DD, Ambrose LG, Browning LN (1995). Trophic dynamics of two sympatric species of riparian spider (Araneae: Tetragnathidae). Can. J. Zool..

[84] Balfour RA, Buddle CM, Rypstra AL, Walker SE, Marshall SD (2003). Ontogenetic shifts in competitive interactions and intra-guild predation between two wolf spider species. Ecol. Entomol..

[85] Travis JMJ (2013). Dispersal and species’ responses to climate change. Oikos.

[86] Travis JMJ (2012). Modelling dispersal: an eco-evolutionary framework incorporating emigration, movement, settlement behaviour and the multiple costs involved. Methods Ecol. Evol..

[87] Bonte D, Lukáč M, Lens L (2008). Starvation affects pre-dispersal behaviour of Erigone spiders. Basic Appl. Ecol..

[88] Goodacre SL (2009). Microbial modification of host long-distance dispersal capacity. BMC Biol..

[89] De Meester N, Bonte D (2010). Information use and density-dependent emigration in an agrobiont spider. Behav. Ecol..

[90] Bonte D, Lens L (2007). Heritability of spider ballooning motivation under different wind velocities. Evol. Ecol. Res..

[91] Clobert J, Galliard J-FL, Cote J, Meylan S, Massot M (2009). Informed dispersal, heterogeneity in animal dispersal syndromes and the dynamics of spatially structured populations. Ecol. Lett..

[92] Titeux N (2020). Ecological traps and species distribution models: a challenge for prioritizing areas of conservation importance. Ecography.

[93] Ceia-Hasse A, Sinervo B, Vicente L, Pereira HM (2014). Integrating ecophysiological models into species distribution projections of European reptile range shifts in response to climate change. Ecography.

[94] Sinervo B (2010). Erosion of lizard diversity by climate change and altered thermal niches. Science.

[95] Slatyer RA, Nash MA, Hoffmann AA (2017). Measuring the effects of reduced snow cover on Australia’s alpine arthropods. Austral Ecol..

[96] Cardoso P (2020). Scientists’ warning to humanity on insect extinctions. Biol. Conserv..

[97] Samways MJ (2020). Solutions for humanity on how to conserve insects. Biol. Conserv..

[98] Prieto-Benítez S, Méndez M (2011). Effects of land management on the abundance and richness of spiders (Araneae): a meta-analysis. Biol. Conserv..

[99] Marc P, Canard A, Ysnel F (1999). Spiders (Araneae) useful for pest limitation and bioindication. Agric. Ecosyst. Environ..

[100] Noss RF, Daly KM, Crooks KR (2006). Incorporating connectivity into broad-scale conservation planning. Connectivity Conservation.

[101] World Conservation Monitoring Centre. The IUCN Red List of Threatened Species 1996 (1996).

[102] Dunnington, D. *ggspatial: Spatial Data Framework for ggplot2*. https://CRAN.R-project.org/package=ggspatial (2020).

[103] Wickham H (2016). ggplot2: elegant graphics for data analysis.

[104] South, A. *rnaturalearth: World Map Data from Natural Earth*. https://CRAN.R-project.org/package=rnaturalearth (2017).

[105] Kassambara, A. *ggpubr: ‘ggplot2’ Based Publication Ready Plots*. https://CRAN.R-project.org/package=ggpubr (2020).

